# Prodromal Parkinson disease signs are predicted by a whole-blood inflammatory transcriptional signature in young *Pink1*^−/−^ rats

**DOI:** 10.1186/s12868-024-00857-0

**Published:** 2024-03-04

**Authors:** Sarah A. Lechner, David G. S. Barnett, Stephen C. Gammie, Cynthia A. Kelm-Nelson

**Affiliations:** 1 Department of Surgery, Division of Otolaryngology-Head and Neck Surgery, Medical Sciences Center, University of Wisconsin–Madison, 1300 University Avenue, 416, Madison, WI 53706 USA; 2 Department of Integrative Biology, University of Wisconsin, Madison, WI USA

**Keywords:** Parkinson disease, Pink1^−/−^, RNA-sequencing, Rat, Whole blood

## Abstract

**Background:**

Parkinson disease (PD) is the fastest growing neurodegenerative disease. The molecular pathology of PD in the prodromal phase is poorly understood; as such, there are no specific prognostic or diagnostic tests. A validated *Pink1* genetic knockout rat was used to model early-onset and progressive PD. Male *Pink1*^−/−^ rats exhibit progressive declines in ultrasonic vocalizations as well as hindlimb and forelimb motor deficits by mid-to-late adulthood. Previous RNA-sequencing work identified upregulation of genes involved in disease pathways and inflammation within the brainstem and vocal fold muscle. The purpose of this study was to identify gene pathways within the whole blood of young *Pink1*^−/−^ rats (3 months of age) and to link gene expression to early acoustical changes. To accomplish this, limb motor testing (open field and cylinder tests) and ultrasonic vocalization data were collected, immediately followed by the collection of whole blood and RNA extraction. Illumina^®^ Total RNA-Seq TruSeq platform was used to profile differential expression of genes. Statistically significant genes were identified and Weighted Gene Co-expression Network Analysis was used to construct co-expression networks and modules from the whole blood gene expression dataset as well as the open field, cylinder, and USV acoustical dataset. ENRICHR was used to identify the top up-regulated biological pathways.

**Results:**

The data suggest that inflammation and interferon signaling upregulation in the whole blood is present during early PD. We also identified genes involved in the dysregulation of ribosomal protein and RNA processing gene expression as well as prion protein gene expression.

**Conclusions:**

These data identified several potential blood biomarkers and pathways that may be linked to anxiety and vocalization acoustic parameters and are key candidates for future drug-repurposing work and comparison to human datasets.

**Supplementary Information:**

The online version contains supplementary material available at 10.1186/s12868-024-00857-0.

## Background

Parkinson disease (PD) is the fastest growing neurological disorder and affects approximately 1% of the world’s population over the age of 60 [1]. The neuropathological hallmark of PD is death of dopaminergic neurons in the substantia nigra which leads to the diagnostic motor signs of the disease (*eg.*, bradykinesia, rigidity, and resting tremor). In most cases, however, a protracted period of significant neuron death, beginning in the prodromal stage, precedes clinical presentation of motor signs [2–4]. Prodromal and early-stage PD is associated with a multitude of heterogeneous non-motor signs and symptoms such as sleep and vision disturbances, olfactory and gastrointestinal dysfunction, anxiety, and early-onset cranial sensorimotor impairments that likely have a variety of systemic pathologies [5]. Moreover, there is no specific biomarker test to diagnose PD in the early-stage, before the cardinal motor features of the disease appear, and consequently diagnosis and treatment are delayed.

Early-stage PD is difficult to investigate in humans due to inconsistencies in symptom manifestation, age of onset, and environmental factors. Biomarker identification research has generally focused on cerebrospinal fluid (CSF), yet CSF collection via lumbar puncture is invasive, expensive, painful, and requires skilled healthcare providers to collect specimens. Alternatively, blood collection via phlebotomy is a ubiquitous, relatively non-invasive source for potential biomarkers that is inexpensive, routine, and still has a high clinical application. Several recent studies have evaluated whole-blood transcriptome data that have demonstrated consistent PD-specific changes in neutrophil gene expression and lymphocyte cell counts linked to the motor progression of PD [6, 7]. Data also suggest that PD gene expression signals such as phosphorylated α-synuclein, DJ-1, and oxidative stress markers are detectable in blood and plasma of clinically-ill patients [8–10]. These studies validate use of peripheral whole blood gene expression in biomarker discovery and potential development of diagnostics and prospective therapeutics. Despite these promising findings, identification and understanding of early-stage PD genetic biomarkers within whole blood samples remains limited.

Most cases of PD are idiopathic; however, 5–10% of cases are monogenic familial forms such as those due to mutations in *LRRK2*, *PRKN*, *DJ1*, *SNCA,* and *PINK1* [11–13]. Large genome-wide association studies have shown these specific genes are concomitantly implicated in idiopathic PD pathophysiology [14], and are involved in a set of molecular pathways that trigger an early-onset pathology sequence that is indistinguishable from sporadic forms [12]. Mitochondria within the CNS are subject to the immense metabolic demands of neuronal activity. Mitochondrial stress, abnormal mitophagy, and lysosomal dysfunction leads to the release of damage-associated molecules that can activate an innate immune response, as seen in genetic murine models of PD. For example, PINK1 (PARK6, phosphatase and tensin homolog (PTEN)-induced putative kinase 1) is involved in mitochondrial quality control and protects cells from stress-induced mitochondrial dysfunction. Altered mitophagy due to Pink1 deficiency is likely involved in multiple CNS disorders, including PD, Alzheimer’s disease, and glaucoma [15]. In general, loss of *Pink1* is also involved in increased generation of pro-inflammatory cytokines and chemokines within plasma, sera, CSF, and blood linked to neuron death [16] as well as increased cytosolic mitochondrial mtDNA and induction of type-I interferon responses and apoptosis [17].

Genetic rodent models of early-stage PD provide insight into the underlying genetics of idiopathic disease, provide experimental control to link genes to behavioral dysfunction associated with disease progression, and identify targets for the development of treatments [18]. The *Pink1*^−/−^ rat parallels human idiopathic PD progression including early-stage behavioral changes due to sensorimotor and cranial motor dysfunction (*eg*, vocal communication) [19, 20]. Work over the past decade has demonstrated that *Pink1*^*−/−*^ rats develop early motor and non-motor deficits as soon as two months of age [21, 22]. This includes alterations in early affected peripheral head and neck muscles and nerves involved in communication, as well as decreased norepinephrine, abnormal α-synuclein aggregation, and increased inflammation in multiple brainstem regions including those associated with sensorimotor vocal function (e*g,* periaqueductal gray; vocal modulator) [22–30]. Rats communicate during social interactions by producing ultrasonic vocalizations (USVs) in part by contraction and adduction of the thyroarytenoid (TA) muscle [31, 32]. At 2–3 months of age, in the prodromal period, *Pink1*^−/−^ rats show differences in acoustical parameters compared to wild-type rats [22, 29]. Recent research has shown that loss of functional Pink1 in the TA muscle leads to increased inflammatory and cell death pathways including the TNF-α/NF-kB signaling pathway [28]. The identification of key disease-related genes and biological pathways are important to develop to identify treatment targets for early signs including communication dysfunction. Yet, there is a need to establish translatability between biomarker identification within tissue types and an easily accessible, comprehensive sample type.

While previous cranial motor behavior and tissue-specific genetic studies have been done in the *Pink1*^*−/−*^ rat model, whole blood gene expression has not been evaluated [25, 28, 29, 33]. By identifying whole blood gene expression profiles in early-stage PD, we can develop a transcriptomic signature capable of detecting PD in prodromal stages. The purpose of the current study was to identify dysregulated gene pathways within the blood of young *Pink1*^*−/−*^ rats (3 months of age), develop genetic biomarkers or signatures that appear during the early-stage of disease, prior to the onset of hallmark limb motor signs, and evaluate whether they can predict early-stage, cranial motor-based vocalization outcomes. Here, we tested the specific hypothesis that loss of *Pink1* alters inflammation gene expression in whole blood, resulting in the upregulation of genetic pathways that begin in early-stage disease and are bioinformatically correlated to vocal communication acoustic parameters.

## Results

### Overall DEG analysis

There were 101 differentially expressed genes (DEG) identified in this study including 16 downregulated and 85 upregulated in *Pink1*^−/−^ compared to WT male rats. *Pink1* was confirmed to be significantly downregulated in the dataset (log(Fold Change) = − 7.49 × 10^10^). Within the entire DEG dataset, the top gene biological processes from the KEGG 2021 Human included mitophagy, ubiquitin mediated proteolysis, apoptosis, PPAR signaling, and others including Parkinson disease (Table 1).

**Table 1:** Gene enrichment KEGG analysis of the DEG dataset

KEGG 2021 human	P-value	Combined score	Genes
Mitophagy	0.005077	48.84843934	PINK1;UBC;ULK1
Ubiquitin mediated proteolysis	0.005756	30.5893287	UBE2H;UBC;NEDD4L;BTRC
Apoptosis	0.006049	29.85283778	PIDD1;TUBA1C;GADD45A;CTSW
PPAR signaling pathway	0.006424	42.71533574	ACADL;ACSL1;UBC
Peroxisome	0.008523	36.22486703	NOS2;ACSL1;ECH1
Pathways of neurodegeneration	0.010884	13.82892126	PRNP;TUBA1C;PINK1;NOS2;UBC;ULK1;COX5B
Ferroptosis	0.018559	40.60210583	PRNP;ACSL1
Fatty acid degradation	0.020303	37.7473815	ACADL;ACSL1
Cell cycle	0.025616	18.15012594	CDKN2C;GADD45A;BUB1
Arginine and proline metabolism	0.026918	29.89881632	NOS2;CKB
Lysosome	0.027792	17.17490197	LAPTM4B;ENTPD4;CTSW
Pyrimidine metabolism	0.033181	25.03114045	ENTPD4;TK1
Amyotrophic lateral sclerosis	0.03869	9.123903065	TUBA1C;PINK1;NOS2;ULK1;COX5B
Parkinson disease	0.038692	10.64788748	TUBA1C;PINK1;UBC;COX5B
Adipocytokine signaling pathway	0.04845	17.92029129	ACSL1;AGRP

ACADL, Acyl-CoA Dehydrogenase Long Chain; ACSL1, Acyl-CoA Synthetase Long Chain Family Member 1; AGRP, Agouti Related Neuropeptide; BTRC, Beta-Transducin Repeat Containing E3 Ubiquitin Protein Ligase; BUB1, BUB1 Mitotic Checkpoint Serine/Threonine Kinase; CDKN2C, Cyclin Dependent Kinase Inhibitor 2C; CKB, Creatine Kinase B; COX5B, Cytochrome C Oxidase Subunit 5B; CTSW, Cathepsin W; ECH1, Enoyl-CoA Hydratase 1; ENTPD4, Ectonucleoside Triphosphate Diphosphohydrolase 4; GADD45A, Growth Arrest And DNA Damage Inducible Alpha; LAPTM4B, Lysosome-associated transmembrane protein 4B; NEDD4L, NEDD4 Like E3 Ubiquitin Protein Ligase; NOS2, Nitric Oxide Synthase 2; PIDD1, P53-Induced Death Domain Protein 1; PINK1, PTEN Induced Kinase 1; PRNP, Major prion protein; TK1, Thymidine Kinase 1; TUBA1C, Tubulin Alpha 1c; UBC, ubiquitin C; UBE2H, Ubiquitin Conjugating Enzyme E2 H; ULK1, Unc-51 Like Autophagy Activating Kinase 1

### Downregulated gene pathways

Downregulated pathways identified using WikiPathways (Table 2) included those related to mitochondrial long-chain fatty acid beta-oxidation (*Acadl*), type II interferon signaling (*Nos2*), and PD (*Pink1*). Within the significantly downregulated DEG dataset, gene enrichment analysis for the GO biological process (Table 3) included nucleotide biosynthesis (*Pink1, Nos2*), apoptotic processes (*Pink1, Lig4*), and immune responses (*Nos2, Lcn2, Mid2*) were identified. Drug compounds identified to reverse downregulated gene transcription (Table 4) included multiple interferon-like compounds (IFNA-MCF7, BT20, SKBR3, MDAB231, HS578T).

**Table 2:** Downregulated gene pathways

WikiPathways 2019	P-value	Combined score	Genes
Mitochondrial LC-fatty acid beta-oxidation WP401	0.013518558	358.0671609	ACADL
Type II interferon signaling (IFNG) WP1253	0.028521354	134.4018728	NOS2
Fatty acid beta oxidation WP1269	0.028521354	134.4018728	ACADL
Parkinsons disease pathway WP3638	0.032650532	112.2527604	PINK1

ACADL, Acyl-CoA Dehydrogenase Long Chain; NOS2, Nitric Oxide Synthase 2; Pink1, PTEN induced putative kinase 1

**Table 3:** Gene enrichment GO Biological Process analysis of the significantly downregulated genes

GO biological processes 2021	P-value	Combined score	Genes
Positive regulation of purine nucleotide biosynthetic process (GO:1900373)	5.27E−05	2384.526542	PINK1;NOS2
Negative regulation of neuron apoptotic process (GO:0043524)	0.001632573	246.9566012	PINK1;LIG4
Innate immune response (GO:0045087)	0.001981189	87.80293162	NOS2;LCN2;MID2
Positive regulation of autophagy (GO:0010508)	0.00260634	179.3506662	PINK1;MID2
Positive regulation of cold-induced thermogenesis (GO:0120162)	0.003019429	161.9703169	ACADL;LCN2
Regulation of neuron apoptotic process (GO:0043523)	0.003080796	159.7193234	PINK1;LIG4
Negative regulation of neuron death (GO:1901215)	0.003080796	159.7193234	PINK1;LIG4
Positive regulation of metabolic process (GO:0009893)	0.004071108	131.3781523	ACADL;LCN2

ACADL, Acyl-CoA Dehydrogenase Long Chain; LCN2, Lipocalin 2; LIG4, DNA Ligase 4; MID2, Midline 2; NOS2, Nitric Oxide Synthase 2; Pink1, PTEN-induced kinase 1

**Table 4:** Drug repurposing using LINCS L1000 and downregulated gene targets

Drug compound	P-value	Combined score	Genes
IFNA-MCF7	2.26E−04	254.9535019	PHF11;LCN2;HERC6
IFNA-BT20	0.005922153	100.5446808	PHF11;HERC6
IFNA-SKBR3	0.006006249	99.52598888	PHF11;HERC6
IFNA-MDAMB231	0.006877312	90.20902738	PHF11;HERC6
IFNA-HS578T	0.006967409	89.35667103	PHF11;HERC6
PDGFBB-SKBR3	0.007519304	84.50918235	LAPTM4B;LCN2
BTC-MCF10A	0.008090459	80.07848334	LAPTM4B;LCN2

Gene HERC6, HECT And RLD Domain Containing E3 Ubiquitin Protein Ligase Family Member 6; LAPTM4B, Lysosome-associated transmembrane protein 4B; PHF11, PHD Finger Protein 11; LCN2, Lipocalin 2

### Upregulated gene pathways

There were notably more upregulated DEG compared to downregulated; this is consistent with DEG expressed in the brain and thyroarytenoid muscle of the *Pink1*^−/−^ rat [25, 28]. The gene enrichment for the gene ontology (GO) biological processes (Table 5) included a long list of cellular activities (cell cycle, ubiquitin activity, protein modifiers) as well as multiple blood-specific processes. These findings were mirrored in the WikiPathways analysis in which fatty acid biosynthesis (*Acsl1, Ech1*), cell cycle control (*Cdkn2c, Gadd45A*), p54 signaling (*Pidd1, Gadd45A*), and PPAR signaling (*Acsl1, Ubc*) were significantly upregulated (Table 6). Finally, drugs that were matched to reverse upregulation included EPG-MCF7, MCF-MCF7 (apoptosis resistance), HBEGF-SKRB3 (Anti-EGFR monoclonal antibodies), and IFNG-MCF7 (interferon) (Table 7).

**Table 5:** Gene enrichment GO biological process analysis of the significantly upregulated genes

GO biological processes 2021	P-value	Combined score	Genes
Mitotic nuclear division (GO:0140014)	2.79E−04	114.5941314	GOLGA2;TPX2;KIF18B;KIFC1
Mitotic spindle organization (GO:0007052)	5.53E−04	60.9428395	GOLGA2;TPX2;STIL;KIFC1;BUB1
Positive regulation of coagulation (GO:0050820)	7.86E−04	428.6577674	DMTN;ENPP4
Positive regulation of hemostasis (GO:1,900,048)	7.86E−04	428.6577674	DMTN;ENPP4
Mitotic spindle assembly (GO:0090307)	8.65E−04	125.0712864	GOLGA2;TPX2;KIFC1
Positive regulation of ubiquitin protein ligase activity (GO:1,904,668)	0.001145981	324.7869518	GOLGA2;BTRC
Regulation of cyclin-dependent protein kinase activity (GO:1,904,029)	0.001571551	91.9912981	CDKN2C;GADD45A;CCNF
Modification-dependent protein catabolic process (GO:0019941)	0.001669146	40.21420442	UBE2H;UHRF1;UBC;NEDD4L;BTRC
Protein modification by small protein conjugation (GO:0032446)	0.001782978	27.57203266	UBE2H;UHRF1;CCNF;UBC;NEDD4L;BTRC;TRIM31
Positive regulation of blood coagulation (GO:0030194)	0.002329057	193.7985609	DMTN;ENPP4
Negative regulation of cation transmembrane transport (GO:1,904,063)	0.002612975	178.2298835	SLC43A1;NEDD4L
Regulation of spindle organization (GO:0090224)	0.002612975	178.2298835	TPX2;TACC3
Negative regulation of dephosphorylation (GO:0035305)	0.003227035	152.7884929	PPP1R15A;SH2D4A
Microtubule nucleation (GO:0007020)	0.003227035	152.7884929	GOLGA2;TPX2
Calcium-mediated signaling using intracellular calcium source (GO:0035584)	0.003556911	142.2838495	PRNP;DMTN
Negative regulation of peptidyl-serine phosphorylation (GO:0033137)	0.003556911	142.2838495	DMTN;GADD45A
Regulation of ubiquitin protein ligase activity (GO:1,904,666)	0.003556911	142.2838495	GOLGA2;BTRC
Ubiquitin-dependent protein catabolic process via the multivesicular body sorting pathway (GO:0043162)	0.003901842	132.9442998	UBAP1;VPS25
Lipid translocation (GO:0034204)	0.004636346	117.0882506	SLC66A2;ABCA4
Regulation of cyclin-dependent protein serine/threonine kinase activity (GO:0000079)	0.005152359	48.39549715	CDKN2C;GADD45A;CCNF
Spindle assembly (GO:0051225)	0.00550998	46.59461952	GOLGA2;TPX2;KIFC1
Regulation of blood coagulation (GO:0030193)	0.005847771	98.57186344	DMTN;ENPP4
Stress-activated protein kinase signaling cascade (GO:0031098)	0.005847771	98.57186344	UBC;BTRC
Negative regulation of phosphatase activity (GO:0010923)	0.006280312	93.46052238	PPP1R15A;SH2D4A
Regulation of protein targeting to membrane (GO:0090313)	0.00672701	88.77492914	PRNP;DMTN
Protein ubiquitination (GO:0016567)	0.006991208	16.67867098	UBE2H;UHRF1;CCNF;UBC;NEDD4L;BTRC;TRIM31
Microtubule polymerization (GO:0046785)	0.007187738	84.46633049	GOLGA2;TPX2
Phospholipid translocation (GO:0045332)	0.007662373	80.49300114	SLC66A2;ABCA4
Positive regulation of ubiquitin-protein transferase activity (GO:0051443)	0.007662373	80.49300114	GOLGA2;BTRC
Regulation of centrosome duplication (GO:0010824)	0.008150791	76.81903446	STIL;CCNF
Organic acid transport (GO:0015849)	0.008907728	35.28697393	SLC16A1;ACSL1;SLC43A1
Organelle disassembly (GO:1,903,008)	0.009168483	70.24902579	GOLGA2;ULK1
Sister chromatid segregation (GO:0000819)	0.009168483	70.24902579	KIF18B;KIFC1
Protein destabilization (GO:0031648)	0.009168483	70.24902579	PRNP;BTRC
Calcium-mediated signaling (GO:0019722)	0.009401506	34.17553607	PRNP;DMTN;CCRL2
Regulation of mitotic spindle organization (GO:0060236)	0.009697515	67.30243038	TPX2;TACC3
Regulation of T cell receptor signaling pathway (GO:0050856)	0.009697515	67.30243038	PRNP;KCNN4
Protein polyubiquitination (GO:0000209)	0.010908959	17.91732573	UBE2H;CCNF;UBC;NEDD4L;BTRC
Regulation of phosphoprotein phosphatase activity (GO:0043666)	0.011363906	59.57463692	PPP1R15A;NUAK1
Regulation of protein serine/threonine kinase activity (GO:0071900)	0.011819423	29.77767145	CDKN2C;GADD45A;CCNF
Mitotic cell cycle phase transition (GO:0044772)	0.012146854	20.94178916	CDKN2C;CCNF;TACC3;BTRC
Positive regulation of wound healing (GO:0090303)	0.012539725	55.19243818	DMTN;ENPP4
Endosome organization (GO:0007032)	0.013146749	53.1940064	PHETA2;ALS2CL
Regulation of viral transcription (GO:0046782)	0.013146749	53.1940064	SUPT4H1;TRIM31
Vesicle organization (GO:0016050)	0.0163684	44.75221974	PHETA2;ALS2CL
Microtubule cytoskeleton organization involved in mitosis (GO:1,902,850)	0.017280771	23.50565989	STIL;TACC3;BUB1
Ubiquitin-dependent protein catabolic process (GO:0006511)	0.017498354	14.17570879	UBE2H;UHRF1;NEDD4L;UBAP1;BTRC
Negative regulation of transcription, DNA-templated (GO:0045892)	0.019131137	9.468307834	UHRF1;GADD45A;UBC;SUPT4H1;DPF3;NEDD4L;BTRC;BAP1;DEDD2
Protein homotetramerization (GO:0051289)	0.020629831	37.1548944	GOLGA2;TK1
Negative regulation of anion transmembrane transport (GO:1,903,960)	0.021072075	228.7280021	SLC43A1
Protein transport to vacuole involved in ubiquitin-dependent protein catabolic process via the multivesicular body sorting pathway (GO:0043328)	0.021072075	228.7280021	VPS25
Pyrimidine ribonucleotide metabolic process (GO:0009218)	0.021072075	228.7280021	ENTPD4
Negative regulation of collateral sprouting (GO:0048671)	0.021072075	228.7280021	ULK1
Regulation of caveolin-mediated endocytosis (GO:2,001,286)	0.021072075	228.7280021	NEDD4L
Regulation of chloride transport (GO:2,001,225)	0.021072075	228.7280021	PRNP
Negative regulation of PERK-mediated unfolded protein response (GO:1,903,898)	0.021072075	228.7280021	PPP1R15A
Regulation of DNA topoisomerase (ATP-hydrolyzing) activity (GO:2,000,371)	0.021072075	228.7280021	UHRF1
Establishment of protein localization to vacuole (GO:0072666)	0.021072075	228.7280021	VPS25
Ethanolamine-containing compound metabolic process (GO:0042439)	0.021072075	228.7280021	GDE1
Exocyst localization (GO:0051601)	0.021072075	228.7280021	EXOC3
Positive regulation of DNA topoisomerase (ATP-hydrolyzing) activity (GO:2,000,373)	0.021072075	228.7280021	UHRF1
Positive regulation of feeding behavior (GO:2,000,253)	0.021072075	228.7280021	AGRP
Modulation by symbiont of host autophagy (GO:0075071)	0.021072075	228.7280021	ULK1
N-acylethanolamine metabolic process (GO:0070291)	0.021072075	228.7280021	GDE1
Regulation of potassium ion transmembrane transport (GO:1,901,379)	0.022142345	35.07102076	NEDD4L;KCNN4
Interstrand cross-link repair (GO:0036297)	0.022915373	34.09768809	DCLRE1B;UBC
DNA damage response, signal transduction by p53 class mediator resulting in cell cycle arrest (GO:0006977)	0.02369944	33.16639759	PIDD1;GADD45A
Protein K48-linked ubiquitination (GO:0070936)	0.02449444	32.27464574	UBE2H;NEDD4L
Pteridine-containing compound biosynthetic process (GO:0042559)	0.025233543	174.4296658	SPR
Purine ribonucleoside catabolic process (GO:0046130)	0.025233543	174.4296658	ENPP4
Amino-acid betaine transport (GO:0015838)	0.025233543	174.4296658	SLC22A5
Regulation of anion channel activity (GO:0010359)	0.025233543	174.4296658	PRNP
Carnitine transport (GO:0015879)	0.025233543	174.4296658	SLC22A5
Negative regulation of protein targeting to membrane (GO:0090315)	0.025233543	174.4296658	DMTN
Regulation of glutamate receptor signaling pathway (GO:1,900,449)	0.025233543	174.4296658	PRNP
Centromeric sister chromatid cohesion (GO:0070601)	0.025233543	174.4296658	BUB1
Regulation of long-chain fatty acid import across plasma membrane (GO:0010746)	0.025233543	174.4296658	ACSL1
Positive regulation of protein glycosylation (GO:0060050)	0.025233543	174.4296658	GOLGA2
Maintenance of DNA methylation (GO:0010216)	0.025233543	174.4296658	UHRF1
Metaphase/anaphase transition of cell cycle (GO:0044784)	0.025233543	174.4296658	TACC3
Metaphase/anaphase transition of mitotic cell cycle (GO:0007091)	0.025233543	174.4296658	TACC3
Monoubiquitinated protein deubiquitination (GO:0035520)	0.025233543	174.4296658	BAP1
Negative regulation of amino acid transport (GO:0051956)	0.025233543	174.4296658	SLC43A1
Phospholipid transport (GO:0015914)	0.026116823	30.60067511	SLC66A2;ABCA4
Selective autophagy (GO:0061912)	0.026116823	30.60067511	UBC;ULK1
Stress-activated MAPK cascade (GO:0051403)	0.028629802	28.33369953	UBC;BTRC
Purine ribonucleoside metabolic process (GO:0046128)	0.029377529	139.3442448	ENPP4
Creatine metabolic process (GO:0006600)	0.029377529	139.3442448	CKB
Regulation of myosin-light-chain-phosphatase activity (GO:0035507)	0.029377529	139.3442448	NUAK1
Piecemeal microautophagy of the nucleus (GO:0034727)	0.029377529	139.3442448	ULK1
Positive regulation by symbiont of host autophagy (GO:0075044)	0.029377529	139.3442448	ULK1
Golgi disassembly (GO:0090166)	0.029377529	139.3442448	GOLGA2
Response to host defenses (GO:0052200)	0.029377529	139.3442448	UBC
Positive regulation of integrin-mediated signaling pathway (GO:2,001,046)	0.029377529	139.3442448	DMTN
Late nucleophagy (GO:0044805)	0.029377529	139.3442448	ULK1
Lysosomal microautophagy (GO:0016237)	0.029377529	139.3442448	ULK1
Modulation by symbiont of host defense response (GO:0052031)	0.029377529	139.3442448	UBC
Protection from non-homologous end joining at telomere (GO:0031848)	0.029377529	139.3442448	DCLRE1B
DNA metabolic process (GO:0006259)	0.030445636	12.4064909	DCLRE1B;UHRF1;GADD45A;UBC
Mitotic G1 DNA damage checkpoint signaling (GO:0031571)	0.03123562	26.31896204	PIDD1;GADD45A
Actin filament reorganization (GO:0090527)	0.033504106	114.9818124	DMTN
Negative regulation of cAMP-dependent protein kinase activity (GO:2,000,480)	0.033504106	114.9818124	PKIA
Regulation of collateral sprouting (GO:0048670)	0.033504106	114.9818124	ULK1
Regulation of dendritic spine maintenance (GO:1,902,950)	0.033504106	114.9818124	PRNP
Negative regulation of potassium ion transmembrane transporter activity (GO:1,901,017)	0.033504106	114.9818124	NEDD4L
Negative regulation of sodium ion transport (GO:0010766)	0.033504106	114.9818124	NEDD4L
Dendritic spine maintenance (GO:0097062)	0.033504106	114.9818124	PRNP
Peptidyl-lysine hydroxylation (GO:0017185)	0.033504106	114.9818124	PLOD1
Regulation of positive chemotaxis (GO:0050926)	0.033504106	114.9818124	F7
Regulation of protein glycosylation (GO:0060049)	0.033504106	114.9818124	GOLGA2
Positive regulation of fibroblast migration (GO:0010763)	0.033504106	114.9818124	DMTN
Positive regulation of homotypic cell–cell adhesion (GO:0034112)	0.033504106	114.9818124	DMTN
Positive regulation of isomerase activity (GO:0010912)	0.033504106	114.9818124	UHRF1
Positive regulation of meiotic nuclear division (GO:0045836)	0.033504106	114.9818124	STRA8
Lipid import into cell (GO:0140354)	0.033504106	114.9818124	ACSL1
Meiotic sister chromatid cohesion (GO:0051177)	0.033504106	114.9818124	BUB1
TORC1 signaling (GO:0038202)	0.033504106	114.9818124	NPRL3
Water homeostasis (GO:0030104)	0.033504106	114.9818124	NEDD4L
Protein tetramerization (GO:0051262)	0.036715013	22.90291137	GOLGA2;TK1
Regulation of cell cycle (GO:0051726)	0.037449209	10.90090233	PPP1R15A;CDKN2C;GADD45A;BAP1
Quaternary ammonium group transport (GO:0015697)	0.037613345	97.17686109	SLC22A5
Negative regulation of protein activation cascade (GO:2,000,258)	0.037613345	97.17686109	F7
Nucleoside diphosphate catabolic process (GO:0009134)	0.037613345	97.17686109	ENTPD4
Regulation of PERK-mediated unfolded protein response (GO:1,903,897)	0.037613345	97.17686109	PPP1R15A
DNA repair (GO:0006281)	0.038236126	10.75710824	DCLRE1B;GADD45A;UBC;BAP1
Proteolysis involved in cellular protein catabolic process (GO:0051603)	0.039583339	21.44576047	UBC;CTSW
DNA damage response, signal transduction by p53 class mediator (GO:0030330)	0.039583339	21.44576047	PIDD1;GADD45A
RNA processing (GO:0006396)	0.040835786	13.12275501	CELF3;SUPT4H1;DEDD2
Receptor recycling (GO:0001881)	0.041705318	83.65592939	PHETA2
Negative regulation of sodium ion transmembrane transport (GO:1,902,306)	0.041705318	83.65592939	NEDD4L
Negative regulation of sodium ion transmembrane transporter activity (GO:2,000,650)	0.041705318	83.65592939	NEDD4L
Neuron projection maintenance (GO:1,990,535)	0.041705318	83.65592939	PRNP
Positive regulation of behavior (GO:0048520)	0.041705318	83.65592939	AGRP
Positive regulation of circadian rhythm (GO:0042753)	0.041705318	83.65592939	BTRC
Long-chain fatty acid import into cell (GO:0044539)	0.041705318	83.65592939	ACSL1
Telomere maintenance in response to DNA damage (GO:0043247)	0.041705318	83.65592939	DCLRE1B
Modification-dependent macromolecule catabolic process (GO:0043632)	0.041705318	83.65592939	UBC
Modulation by symbiont of host process (GO:0044003)	0.041705318	83.65592939	UBC
Regulation of organelle assembly (GO:1,902,115)	0.042534001	20.1265342	STIL;NPRL3
Positive regulation of apoptotic process (GO:0043065)	0.043157194	9.945815682	PIDD1;PRNP;GADD45A;UBC
Negative regulation of protein serine/threonine kinase activity (GO:0071901)	0.043535419	19.71433284	GADD45A;PKIA
Negative regulation of centrosome duplication (GO:0010826)	0.045780097	73.0775545	CCNF
Pyrimidine nucleoside salvage (GO:0043097)	0.045780097	73.0775545	TK1
Pyrimidine-containing compound salvage (GO:0008655)	0.045780097	73.0775545	TK1
Negative regulation of interleukin-17 production (GO:0032700)	0.045780097	73.0775545	PRNP
Negative regulation of potassium ion transmembrane transport (GO:1,901,380)	0.045780097	73.0775545	NEDD4L
Negative regulation of protein localization to cell surface (GO:2,000,009)	0.045780097	73.0775545	NEDD4L
Negative regulation of protein maturation (GO:1,903,318)	0.045780097	73.0775545	PRNP
Erythrocyte development (GO:0048821)	0.045780097	73.0775545	DMTN
Golgi inheritance (GO:0048313)	0.045780097	73.0775545	GOLGA2
Tetrahydrobiopterin metabolic process (GO:0046146)	0.045780097	73.0775545	SPR
Negative regulation of activated T cell proliferation (GO:0046007)	0.045780097	73.0775545	PRNP
Regulation of mitotic cell cycle phase transition (GO:1,901,990)	0.046095937	12.00589545	TPX2;UBC;BTRC
Negative regulation of protein modification process (GO:0031400)	0.046591985	18.55228225	PPP1R15A;PRNP
Proteasome-mediated ubiquitin-dependent protein catabolic process (GO:0043161)	0.047968823	9.27259786	CCNF;UBC;NEDD4L;BTRC
Vascular transport (GO:0010232)	0.049725133	17.4915333	SLC22A5;SLC16A1
Negative regulation of calcineurin-mediated signaling (GO:0106057)	0.049837753	64.60145816	PRNP
Negative regulation of calcineurin-NFAT signaling cascade (GO:0070885)	0.049837753	64.60145816	PRNP
Regulation of amyloid precursor protein catabolic process (GO:1,902,991)	0.049837753	64.60145816	PRNP
Cell cycle checkpoint signaling (GO:0000075)	0.049837753	64.60145816	BUB1
Regulation of feeding behavior (GO:0060259)	0.049837753	64.60145816	AGRP
Nucleoside salvage (GO:0043174)	0.049837753	64.60145816	TK1
Regulation of sister chromatid cohesion (GO:0007063)	0.049837753	64.60145816	BUB1

ABCA4, ATP Binding Cassette Subfamily A Member 4; ACSL1, Acyl-CoA Synthetase Long Chain Family Member 1; AGRP, Agouti Related Neuropeptide; ALS2CL, ALS2 C-Terminal Like; BAP1, ubiquitin carboxyl-terminal hydrolase; BTRC, Beta-Transducin Repeat Containing E3 Ubiquitin Protein Ligase; BUB1, Mitotic Checkpoint Serine/Threonine Kinase; CDKN2C, Cyclin Dependent Kinase Inhibitor 2C; CELF3, CUGBP Elav-Like Family Member 3; CCNF, Cyclin F; CCRL2, C–C Motif Chemokine Receptor Like 2; CKB, Creatine Kinase B; CTSW, Cathepsin W; DCLRE1B, DNA Cross-Link Repair 1B; DEDD2, Death Effector Domain Containing 2; DMTN, Dematin Actin Binding Protein; DPF3, Double PHD Fingers 3; ENPP4, Ectonucleotide Pyrophosphatase/Phosphodiesterase 4; EXOC3, Exocyst Complex Component 3; F7, Coagulation Factor VII; GADD45A, Growth Arrest And DNA Damage Inducible Alpha; GDE1, Glycerophosphodiester Phosphodiesterase 1; GOLGA2, Golgin A2; KCNN4, Potassium Calcium-Activated Channel Subfamily N Member 4; KIF18B, Kinesin Family Member 18B; KIFC1, Kinesin Family Member C1; NEDD4L, NEDD4 Like E3 Ubiquitin Protein Ligase;; NPRL3, NPR3 Like, GATOR1 Complex Subunit; NUAK1, AMPK-related protein kinase 5; PIDD1, P53-Induced Death Domain Protein 1; PHETA2, PH Domain Containing Endocytic Trafficking Adaptor 2; PKIA, CAMP-Dependent Protein Kinase Inhibitor Alpha; PLOD1, Procollagen-Lysine,2-Oxoglutarate 5-Dioxygenase 1; PPP1R15A, Protein Phosphatase 1 Regulatory Subunit 15A; PRNP, prion protein; SH2D4A, SH2 Domain Containing 4A; SLC16A1, monocarboxylate transporter; SLC22A5, Solute Carrier Family 22 Member 5; SLC43A1, Solute Carrier Family 43 Member 1; SLC66A2, Solute Carrier Family 66 Member 2; SPR, Sepiapterin Reductase; SUPT4H1, SPT4 Homolog, DSIF Elongation Factor Subunit; STRA8, Stimulated By Retinoic Acid 8; STIL, STIL Centriolar Assembly Protein; TACC3, Transforming Acidic Coiled-Coil Containing Protein 3; TK1, Thymidine Kinase 1; TPX2, TPX2 Microtubule Nucleation Factor; TRIM31, Tripartite Motif Containing 31; UBAP1, Ubiquitin Associated Protein 1; UBC, ubiquitin gene; UBE2H, Ubiquitin Conjugating Enzyme E2 H; UHRF1, Ubiquitin Like With PHD And Ring Finger Domains 1); ULK1, Unc-51 Like Autophagy Activating Kinase 1; VPS25, Vacuolar protein-sorting-associated protein 25

**Table 6:** Gene enrichment WikiPathways analysis of significantly upregulated genes

WikiPathways 2019	P-value	Combined score	Genes
Fatty acid biosynthesis WP336	0.003901842	132.9442998	ACSL1;ECH1
G1 to S cell cycle control WP413	0.027781692	29.05924205	CDKN2C;GADD45A
p53 signaling WP2902	0.033023072	25.09708755	PIDD1;GADD45A
Heme biosynthesis WP18	0.037613345	97.17686109	PPOX
PPAR signaling pathway WP2316	0.046591985	18.55228225	ACSL1;UBC

ACSL1, Acyl-CoA Synthetase Long Chain Family Member 1; CDKN2C, Cyclin Dependent Kinase Inhibitor 2C; ECH1, Enoyl-CoA Hydratase 1; GADD45A, Growth Arrest And DNA Damage Inducible Alpha; PIDD1, P53-Induced Death Domain Protein 1; PPOX, protoporphyrinogen oxidase; UBC, ubiquitin gene

**Table 7:** Drug repurposing using LINCS L1000 and upregulated gene targets

Drug compound	P-value	Combined Score	Genes
EPG-MCF7	0.028411517	16.93884769	TSPAN8;PKIA;CKB
MCSF-MCF7	0.030318056	16.20021062	PRNP;TSPAN8;KCNN4
HBEGF-SKBR3	0.034329088	14.85626319	CDKN2C;ACSL1;TROAP
IFNG-MCF7	0.038053691	13.80794649	PRNP;PKIA;KCNN4

ACSL1, Acyl-CoA Synthetase Long Chain Family Member 1; CDKN2C, Cyclin-dependent kinase 4 inhibitor C; CKB, Creatine Kinase B; KCNN4, Potassium Calcium-Activated Channel Subfamily N Member 4; PKIA, CAMP-Dependent Protein Kinase Inhibitor Alpha; PRNP, prion protein; TSPAN8, Tetraspanin 8; TROAP, Trophinin Associated Protein

### Top 1000 genes

To expand the number of pathways and capture significant biological changes in both directions, the top 1000 up- and downregulated genes were used in a separate gene enrichment analysis. Additional file 1: File S1 lists the significant WikiPathways and GO Biological Process for both up- and downregulated gene lists, respectively. Upregulated pathways included heme biosynthesis (*Urod, Uors, Hmbs, Cpox, Ppox*) and ferroptosis (*Prnp; Steap3; Map1lc3a; Tfrc; Acsl1; Lpcat3; Alox15; Slc11a2; Slc3a2*). Downregulated pathways included mitochondrial fatty acid synthesis (*Mecr; Oxsm; Hsd17b12*) and mitochondrial long-chain fatty acid beta-oxidation (*Pecr; Acadl; Cpt2; Eci1*).

**Fig. 1:**
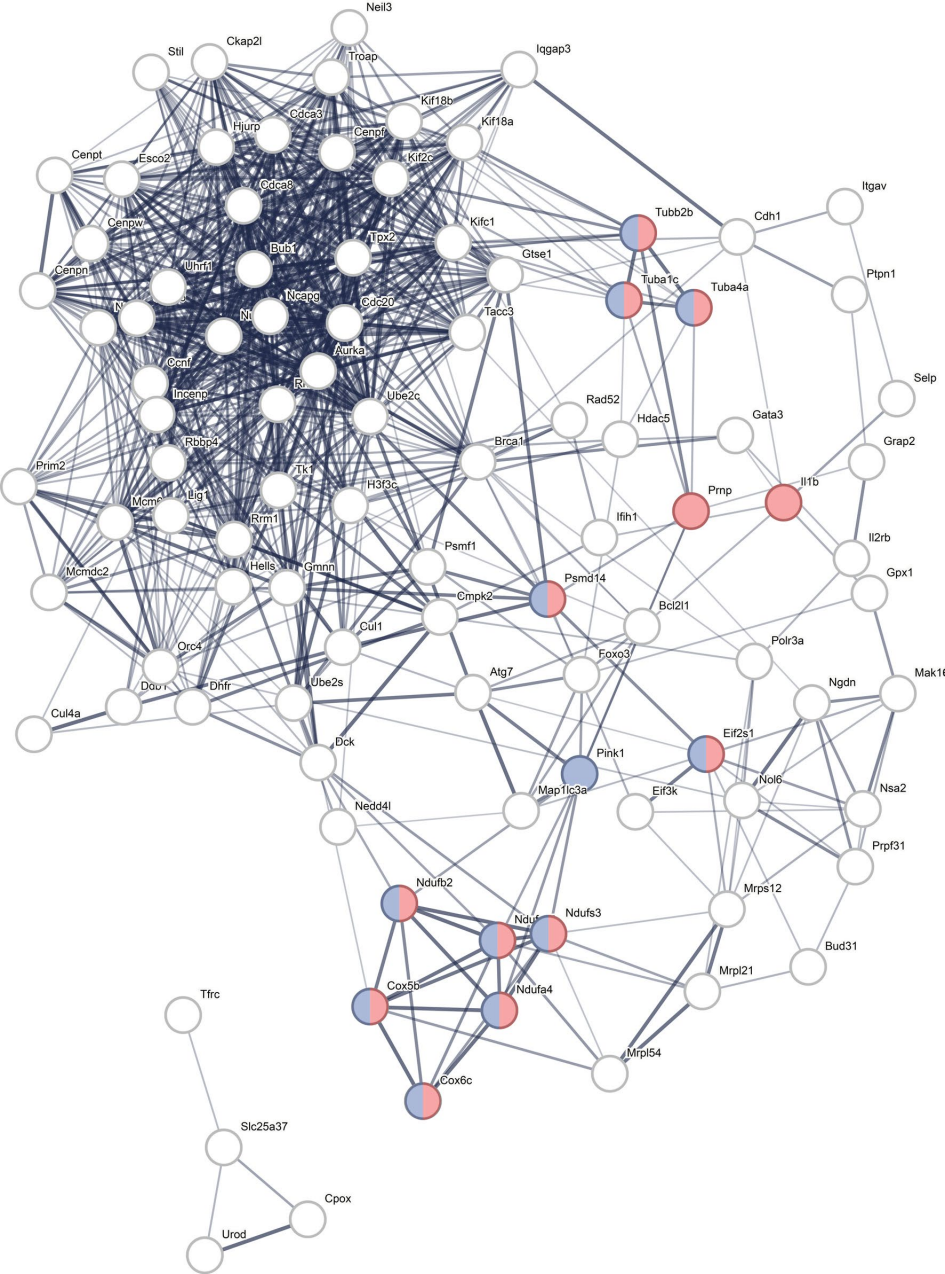
STRING protein–protein interaction map with 97 interconnected genes. Nodes indicate protein and edges indicate protein interactions; line width reflects strength of evidence. Significant enrichment for Parkinson disease (purple) and prion disease (pink) (and overlapping) were identified

When the top 1000 up-and downregulated genes were put in STRING, 96 interacting genes were identified (Additional file 2: File S2). These are replotted and shown in Fig. 1 and demonstrate enrichment for PD and prion disease.

### WGCNA

Additional file 3: File S3 includes the sortable output files, *P*-values, correlations, and list of genes in the top modules. There were 4 significant modules (ME): Red, Yellow, Midnightblue, and Purple. The top module was Red and included *Pink1*. To determine the genes and their functions that interact with Pink1 within this whole blood RNA dataset, the 248 genes that were in Red were put into the gene enrichment analysis tool to evaluate this specific gene list against preexisting data sets. Several areas of enrichment were identified including protein catabolic process, ion homeostasis, and protein destabilization. The Yellow module significantly correlated to frequency modulated USV duration (length) and bandwidth (frequency range) and Yellow (335 genes) was enriched for mitochondrial gene expression. Both the Red and Yellow modules correlated to open field number of entries (movement into the open field indicating less anxiety; increased exploration). Other significant modules, Midnightblue (89 genes) demonstrated enrichment in iron ion homeostasis, as well as macrophage activation, and immune processes. Purple (152 genes) showed enrichment in multiple cellular processes.

## Discussion

The general understanding of inherited, early-onset monogenic forms of PD is limited, yet necessary to provide insight into the polygenic nature of idiopathic PD as well as the development of candidate biomarkers which may be useful in early-stage diagnosis. Whole blood collection is a relatively non-invasive source of potential biomarkers that is inexpensive, easy to obtain, and has translatable clinical relevance. We hypothesized that loss of *Pink1* alters inflammation gene expression in whole blood, resulting in the upregulation of genetic pathways that begin in early-stage disease. Further, we hypothesized that these dysregulated genetic pathways are bioinformatically correlated to behavioral outcomes including motor, anxiety, and vocal communication (cranial motor) acoustic parameters. The *Pink1*^−/−^ rat was selected because it exhibits parallels to human prodromal PD features, including early and progressive changes to vocalization and anxiety with a gradual onset of limb motor dysfunction in adults [29]. The present study identified several dysregulated genes and biological pathways within the blood of young *Pink1*^*−/−*^ rats. These data suggest that the earliest PD signs, independent of nigrostriatal dopamine loss, are bioinformatically correlated to blood pathway data.

### Loss of *Pink1* results in dysregulation of ribosomal protein and RNA processing gene expression

Consistent with previous sequencing studies on brain and TA muscle, there were notably more upregulated than downregulated DEG in *Pink1*^*−/−*^ rats. The most significantly downregulated genes were *Rpl12* (ribosomal protein L12) and *Lyzl1* (Lysozyme like protein 1). *Lzyl1* is a protein coding gene that has been recently identified in a microarray study as a new locus associated with dementia in PD [34]. Some ribosomal proteins, such as ribosomal protein s15, have been linked to neurodegeneration in LRRK2 overexpression human dopamine neuron models [35]. The most significantly upregulated gene in whole blood was *Celf3* (CUGBP Elav-Like Family Member 3). *Celf3* codes for an RNA-binding protein (RBP) involved in various aspects of RNA processing including nucleic acid binding and pre-mRNA alternative splicing. Dysregulation of RBPs has been implicated in neurodegenerative diseases including Alzheimer’s disease (AD), amyotrophic lateral sclerosis (ALS), and PD. RBPs have been found in inclusion bodies of some of these diseases, providing insight into the misfolding of proteins and subsequent protein aggregation. [36–38]

### Interferon signaling is altered in whole blood of ***Pink1***^***−/−***^ rats

Loss of *Pink1* results in increased production of proinflammatory cytokines and chemokines including tumor necrosis factor-α (TNF-α), interleukin-6 (IL-6) and interleukin1-β (IL1-β), as well as interferons (IFNs) IFN-β1 and IFN-γ, within the blood and brain resulting in inflammation and loss of dopaminergic neurons in both a *Pink1*^*−/−*^ mouse model and PINK1-associated PD patients [16]. IFNs exhibit antiproliferative, proapoptotic, antiangiogenic and immunomodulatory functions. In cellular models of PD, loss of Pink1 also increased cytosolic dsDNA derived from mitochondria which resulted in elevated type-I IFN responses and correlated with apoptotic markers and cell death [17].

Type-I IFNs (IFN-Is; IFN-α and IFN-β) play a critical role in the innate immune responses by activating classical proinflammatory signaling pathways that result in the production of major inflammatory cytokines: TNF-α, IL-6, and IL1-β. IFN-Is have been shown to regulate neuroinflammation in the central nervous system (CNS) and contribute to degeneration and disease progression in several in vivo and in vitro models of PD [39, 40]. Mouse models of PD and postmortem studies of PD brains have confirmed that mRNA expression of IFN-Is is upregulated in PD. Whereas IFN-β deficiency causes mitochondrial dysfunction in primary cortical neuron cultures and causes defects in the nigrostriatal dopaminergic pathway as well as widespread α-synuclein accumulation in *Ifnb*^*−/−*^ mice [41]. In addition to Type-I IFNs, Type-II IFNs have also been implicated in PD pathophysiology. For instance, IFN-γ is elevated in the serum and brain of patients with PD and correlates with disease severity [42, 43].

Several genes identified in this dataset included those related to type I interferon signaling (*Ifit1bl*; interferon-induced protein with tetratricopeptide repeats 1B-like), type II interferon signaling (*Nos2*; nitric oxide synthase 2), and apoptosis (*Dedd2*; death effector domain containing 2). Interferon stimulated genes involved in chromatin remodeling (*Gadd45a; Supt4h1; Esco2; Pelp1; Bap1; Tada2a*) and ATP-binding proteins (*Lig4; Slc22a5; Entpd4; Prnp; Vps25; Kifc1; Bub1; Uhrf1; Pidd1; Nek2l1; Abca4; Kif18b; Ckb*) were upregulated in our dataset. Further, unpublished data from our lab show that at 12 months of age, *Pink1*^*−/−*^ rats have significantly more up- and downregulated genes compared to age-matched WT rats (upregulated, *n* = 553; downregulated, *n* = 1561). In this unpublished data set, numerous interferon stimulated genes (ISGs) were upregulated including *Rnasel*, *Fas*, *Casp4*, *Irf1*, and *Ifitm1*. The second most significantly upregulated gene is *Ifit1* (interferon-induced protein with tetratricopeptide repeats 1; *P* = 5.53 × 10^–27^). In addition, receptors for IFN-α (*Ifnar2*) and IFN-γ (*Ifngr1*) were also upregulated in the dataset. Therefore, these data suggest that inflammation and interferon signaling begins early in the whole blood (3 months of age) and progresses as *Pink1*^*−/−*^ rats age. Further work will use bioinformatics to correlate these pathways to behavioral data at 12 months of age.

Drug compounds identified to reverse up- and downregulated gene transcription (Table 4) included multiple interferon-like compounds (IFNA-MCF7, BT20, SKBR3, MDAB231, HS578T and IFNG-MCF7).

From this dataset, we hypothesize that the lack of Pink1 may cause an early disruption in interferon signaling that may result in the downstream overproduction of proinflammatory cytokines (TNF-α, IL-1β, IL-6) that will worsen over time. Targeting interferon signaling with drug compounds may be a potential therapeutic intervention to halt or prevent the further production of harmful proinflammatory cytokines that contribute to neuroinflammation and the death of neurons and should be studied in future work.

### Major prion protein gene expression is upregulated

STRING analysis showed enrichment for prion disease and PD (as in Fig. 1). One of the most interesting genes identified in this dataset was *Prnp*, which was significantly upregulated in *Pink1*^*−/−*^ whole blood as early as 3 months of age. *Prnp* encodes major prion protein (PrP) that is primarily active in the brain and associated with several prion and prion-like diseases. This data further supports the prion hypothesis for PD that has been proposed due to the prion-like misfolding and aggregation of α-synuclein [44–49]. In a prion disease cell model, PINK1/Parkin signaling, specifically PINK1, was required for mitophagy of damaged mitochondria and activation attenuates prion-induced neuronal apoptosis [49]. To our knowledge, this is the first monogenic PD animal model to report significant genetic changes in the *Prnp* gene. In this dataset, *Prnp* was a significant gene in numerous GO Biological Processes identified through gene enrichment of the significantly upregulated genes including dendritic spine maintenance, apoptotic processes, negative regulation of interleukin-17 production, T-cell receptor signaling, and calcium-mediated signaling. There were only four drug compounds identified to reverse upregulated gene transcription and two of them, MCSF-MCF7 and IFNG-MCF7 included *Prnp* as a significant gene.

### ***Tuba1c***, a previously identified significant gene in ***Pink1***^−/−^ rats, is upregulated in whole blood

*Tuba1c* upregulation has been recently identified in several of our RNA-sequencing datasets including the thyroarytenoid (TA) vocal fold muscle and brainstem [29]. Previously, *Tuba1c* was identified as key gene correlated to vocalization acoustic parameter at 2 months of age. In this study, *Tuba1c* was once again significantly upregulated in whole blood of *Pink1*^*−/−*^ rats; it is also an interconnected gene in the STRING analysis. A recent proteomics study, differential expression of Tuba1c protein was identified in the plasma of rotenone-exposed rats [50]. Here, *Tuba1c* was also identified as a significant gene in the gene enrichment KEGG pathway analysis including, apoptosis, pathways of neurodegeneration, ALS, and Parkinson disease (Table 1).

### Bioinformatics analysis highlighted gene pathways that significantly correlate to behavioral outcomes in ***Pink1***^***−/−***^ rats

Another goal of this study was to use bioinformatics to highlight biological gene pathways within the whole blood and determine whether they are significantly correlated to anxiety, motor, or ultrasonic vocalization behavioral outcomes in *Pink1*^*−/−*^ rats. WGCNA enrichment analysis resulted in four significant modules, in which two of the four modules (Red and Yellow) were significantly correlated to behavioral outcomes. The Red module, which contained the *Pink1* gene, correlated to open field number of entries (movement into the open field indicating less anxiety; increased exploration) (Table 8). The Red module demonstrated enrichment in the most biological processes including cell division, chromatin organization, regulation of autophagy, cellular response to ATP and reactive oxygen species, and regulation of the cell cycle. The Yellow module significantly correlated to frequency modulated USV duration (length) and bandwidth (frequency range) as well as open field number of entries. Enrichment of the Yellow module included mitochondrial gene expression, lipid transport across blood–brain barrier, tRNA processing, and IL-7 signaling.

**Table 8:** WGCNA modules and correlated behavioral variables

Significant WGCNA module	Significant behavioral variables		
	Cylinder	Open field	FM USV
Red	N/a	# Entries	N/a
Yellow	N/a	# Entries	Duration (average, maximum, average of the top 10 calls) Bandwidth (average, maximum, average of the top 10 calls)
Midnightblue	N/a	N/a	N/a
Purple	N/a	N/a	N/a

### Limitations

The *Pink1*^−/−^ rat is a useful model to study aspects of early-stage PD, including cranial sensorimotor dysfunction in the absence of nigrostriatal dopamine loss [20]. However, it should be noted that there have been inconsistencies between research groups reporting number of nigral neurons as well as striatal dopamine concentrations in older *Pink1*^−/−^ male rats [51]. Genetic drift, variation among cohorts of animals, differences in experimental and housing paradigms, and other laboratory variables are all common factors in producing inconsistent results and should be considered when making conclusions. The *Pink1*^*−/−*^ rat is a progressive model, and much of the neurological quantification has been done in late adulthood (8–12 months); when emphasizing prodromal behavior and neurochemical future studies should target younger prodromal ages (i.e. 2–3 months). Likewise, it would be interesting to compare *Pink1*^−/−^ transcription with a focus on mitochondrial dysfunction to other PD genetic models, overexpression (synuclein) or neurotoxin lesion. In this study, due to smaller sample sizes used in RNA-seq it is not possible to correlate finding to behavioral outputs. The utility of these findings is the focus on annotated protein coding mRNA and biological pathways for future study. Future work could involve the analysis of non-coding RNA as well as comparison to existing human datasets [52]. While this study focuses on mRNA that encodes protein, non-coding RNA can regulate cellular functions and signal transduction [53]. This study provides the targets for additional studies that focus on particular biological gene networks and manipulation. An additional limitation of this work is that females were not included, and future work should evaluate sex as a biological variable.

## Conclusions

Neuroimaging and CSF biomarkers may be useful in research settings, but due to the ease, availability, and low cost of phlebotomy**,** whole blood biomarkers are among the most promising and practical methods to screen large populations for an occult, yet common and devastating disease with accelerating incidence. Whole blood genetic biomarkers of PD hold promise to screen large populations for PD risk factors. They may also inform prognosis as well as monitor response to future disease-modifying treatments of PD applied in the early-stage of disease, prior to manifestation of hallmark motor signs that currently form the basis of diagnosis. Using validated, monogenic rat models we can study the influence of Parkinsonian genes and their networks and provide data that is translatable to humans. PD has many different identified genes and pathways—mitochondrial dysfunction, deranged immune responses, oxidative stress, and prion protein. This study demonstrates that we can identify PD signature prior to development of a clinical motor phenotype and predict progression of ultrasonic vocalization parameters. Thus, using bioinformatics and whole blood sampling, it may be possible to identify genetic signatures in humans that correlate to vocalization dysfunction and target these gene signatures therapeutically for the treatment of vocal deficits in PD.

## Methods

### Animals and experimental design

A total of 4 male Long-Evans rats with homozygous *Pink1* knockout and 4 male wild-type (WT) Long-Evans control rats (Inotiv, Chicago, IL, USA), aged 3 months, were used in this study. All rats arrived at 4 weeks old and were pair-housed (same-genotype) in standard polycarbonate cages (17 cm × 28 cm × 12 cm) with corncob bedding. Food and water were provided ad libitum. Following arrival, all rats were immediately placed on a 12:12-h reverse light cycle as rats are nocturnal. All behavioral testing occurred under partial red-light illumination. Rats were acclimated to study procedures and experimenter handling prior to all behavioral testing. All rats were weighed upon arrival and weekly using a digital scale to monitor overall health. All procedures and protocols (M006329) were approved by the University of Wisconsin-Madison School of Medicine and Public Health Animal Care and Use Committee and were conducted in accordance with the NIH Guide for the Care and Use of Laboratory animals (National Institutes of Health, Bethesda, MA, USA).

### Behavior

Corresponding rat behavioral data used in this study included open field (time in center ([sec]), number of entries, total movement [(cm])), cylinder limb motor (number of rears and lands, hindlimb and forelimb movements), and ultrasonic vocalizations (total number of calls, duration ([msec]), bandwidth ([kHz]), intensity [(dB]), and peak frequency ([kHz])). These measures were used in the gene statistical correlation analysis, discussed below, and were previously published by Lechner et al. [29].

### Whole blood collection and RNA processing

Whole blood samples were collected from the body trunk during euthanasia by rapid decapitation via guillotine under heavy isoflurane anesthesia. Approximately 400 µl of trunk blood was immediately transferred to a sterile 2 mL microcentrifuge tube that contained 1.3 mL of RNAlater™ RNA stabilization Solution (Invitrogen, Carlsbad, CA, USA) and inverted several times. RNA extraction was then performed using the Ribopure™ kit, per manufacturer’s instructions (Invitrogen). Briefly, the supernatant was removed, and blood cells were lysed with lysis and sodium acetate solution. An acid-phenol chloroform extraction was performed, and the RNA was purified through a filter cartridge and eluted. Total RNA was measured using a Nanodrop system (Thermo Scientific, Wilmington, DE, USA) as well as with an Agilent RNA 6000 Pico kit (Eukaryote Total RNA Pico, Agilent Technologies, Santa Clara, CA) and the Agilent 2100 bioanalyzer (Agilent Technologies, Santa Clara, CA, USA).

### RNA sequencing

All RNA sequencing procedures followed ENCODE and were performed at the University of Wisconsin Biotechnology Center’s Next Generation Sequencing Facility [25, 28] using the Illumina^®^ HiSeq 2000 high-throughput sequencing system (Illumina Inc., San Diego, CA, USA). The Illumina RiboZero Plus Kit (Cat. 20,040,526) with rRNA and globin reduction was used to remove cytoplasmic and mitochondrial rRNA and a sequencing library was generated. Libraries were quantified using Qubit DNA HS kit, diluted 1:100, and assayed on an Agilent DNA1000 chip. Adaptor sequences, contamination and low-quality reads were removed. Reads were mapped to the annotated *Rattus norvegicus* genome in Ensembl. As reviewed in Kelm-Nelson and Gammie, 2020, the technical quality was determined using multiple parameters [25].

### Gene expression analyses

Gene analysis was performed with the glm using the EdgeR Bioconductor Package, v. 3.9. The *P*-value cutoff was set to 0.05 for significance. All raw data were uploaded to the Gene Expression Omnibus (https://www.ncbi.nlm.nih.gov/geo/query/acc.cgi?acc=GSE213543; GSE213543). The RSEM approach for normalizing RNA seq data was used. EdgeRglm data are provided in Additional file 4: Table S1. Statistically significant differentially expressed genes (DEG) were ranked according to *P*-value, FDR, and sorted by up- or downregulation, GO function, biological process, and component (Additional file 5: Table S2).

EnrichR pathway gene enrichment analysis (KEGG analysis) was used to determine gene enrichment on the DEG list and WikiPathway 2021 and GO Biological process was used on the top 1000 up- and 1000 downregulated genes, respectively (Additional file 6: Table S3). Additionally, the top overall 1000 genes were entered into STRINGdp (v 2.0; Search Tool for the Retrieval of Interacting Genes/Proteins, http://string.embl.de/) to identify protein–protein interactions [54, 55]. The top connected genes were replotted with enrichment.

### Weighted gene co-expression network analysis (WGCNA) and behavior

A WGCNA was used to construct co-expression networks and modules from the whole blood gene expression dataset as well as the open field, cylinder, and USV acoustical dataset, previously published in 2021 [29]. Data were log_2_(x + 1) transformed, low expression genes were removed and WGCNA was run (13,360 number of genes) using R Statistical Software [56]. Correlations were raised to a soft thresholding power β of 12, unsupervised hierarchical clustering for WGCNA used the default setting as well as the following: the minimum module size of 30 genes, the signed TOMType, the deepSplit parameter set to 2, and the mergeCutHeight parameter set to 0.25. Searchable networks were created.

### Supplementary Information

**Additional file 1: File S1.** File containing significant WikiPathways and GO biological processes for both up and downregulated differentially expressed genes, respectively. P-value, combined score, and list of genes in each pathway are included.

**Additional file 2: File S2.** String.

**Additional file 3: File S3.** Files containing sortable WGCNA results, P-values, correlations, and genes in the top modules. Enrichment for GO biological process on each of the significant modules (ME red, midnightblue, purple, and yellow).

**Additional file 4: Table S1.** Results of a differential gene expression. Analysis of RNA-Seq data using EdgeR. A negative logFC.

**Additional file 5: Table S2.** Results of a differential gene expression (DEG; protein coding genes) in the whole blood of male rats.

**Additional file 6: Table S3.** Gene list for the Top 1000 up and downregulated genes.

## Data Availability

The datasets generated and/or analyzed during the current study are available in the NCBI GEO Data repository or available through the corresponding author.

## Abbreviations

AD: Alzheimer disease
ALS: Amyotrophic lateral sclerosis
CNS: Central nervous system
CSF: Cerebrospinal fluid
DEG: Differentially expressed gene
GO: Gene Ontology
IFN: Interferon
NF-kB: Nuclear factor kappa-light-chain-enhancer of activated B cells
PD: Parkinson disease
PINK1: PTEN-induced kinase 1
*Pink1*^−/−^: *Pink1* Homozygous knockout
RBP: RNA binding protein
RNA: Ribonucleic acid
STRING: Search tool for the retrieval of interacting genes/proteins
TA: Thyroarytenoid muscle
TNF-α: Tumor necrosis factor α
USV: Ultrasonic vocalization
WGCNA: Weighted gene co-expression network analysis
WT: Wild-type
